# Heritability and reliability of automatically segmented human hippocampal formation subregions

**DOI:** 10.1016/j.neuroimage.2015.12.039

**Published:** 2015-12-30

**Authors:** Christopher D. Whelan, Derrek P. Hibar, Laura S. van Velzen, Anthony S. Zannas, Tania Carrillo-Roa, Katie McMahon, Gautam Prasad, Sinéad Kelly, Joshua Faskowitz, Greig deZubiracay, Juan E. Iglesias, Theo G.M. van Erp, Thomas Frodl, Nicholas G. Martin, Margaret J. Wright, Neda Jahanshad, Lianne Schmaal, Philipp G. Sämann, Paul M. Thompson

**Affiliations:** a Imaging Genetics Center, University of Southern California, Marina del Rey, CA, USA; b Department of Psychiatry and Neuroscience Campus Amsterdam, VU University Medical Center and GGZ inGeest, Amsterdam, The Netherlands; c Department of Translational Research in Psychiatry, Max Planck Institute of Psychiatry, Munich, Germany; d Department of Psychiatry and Behavioral Sciences, Duke University Medical Center, Durham, NC, USA; e Centre for Advanced Imaging, University of Queensland, Brisbane, Australia; f Faculty of Health, Queensland University of Technology, Brisbane, Australia; g Basque Center on Cognition, Brain and Language, Donostia, Gipuzkoa, Spain; h Department of Psychiatry and Human Behavior, University of California, Irvine, USA; i Department of Psychiatry, Otto-von Guericke-University of Magdeburg, Germany; j QIMR Berghofer Medical Research Institute, Brisbane, Queensland, Australia; k Queensland Brain Institute, University of Queensland, Brisbane, Australia

## Abstract

The human hippocampal formation can be divided into a set of cytoarchitecturally and functionally distinct subregions, involved in different aspects of memory formation. Neuroanatomical disruptions within these subregions are associated with several debilitating brain disorders including Alzheimer’s disease, major depression, schizophrenia, and bipolar disorder. Multi-center brain imaging consortia, such as the Enhancing Neuro Imaging Genetics through Meta-Analysis (ENIGMA) consortium, are interested in studying disease effects on these subregions, and in the genetic factors that affect them. For large-scale studies, automated extraction and subsequent genomic association studies of these hippocampal subregion measures may provide additional insight. Here, we evaluated the test–retest reliability and transplatform reliability (1.5 T *versus* 3 T) of the subregion segmentation module in the *FreeSurfer* software package using three independent cohorts of healthy adults, one young (Queensland Twins Imaging Study, N = 39), another elderly (Alzheimer’s Disease Neuroimaging Initiative, ADNI-2, N = 163) and another mixed cohort of healthy and depressed participants (Max Planck Institute, MPIP, N = 598). We also investigated agreement between the most recent version of this algorithm (v6.0) and an older version (v5.3), again using the ADNI-2 and MPIP cohorts in addition to a sample from the Netherlands Study for Depression and Anxiety (NESDA) (N = 221). Finally, we estimated the heritability (*h*^2^) of the segmented subregion volumes using the full sample of young, healthy QTIM twins (N = 728). Test–retest reliability was high for all twelve subregions in the 3 T ADNI-2 sample (intraclass correlation coefficient (ICC) = 0.70–0.97) and moderate-to-high in the 4 T QTIM sample (ICC = 0.5–0.89). Transplatform reliability was strong for eleven of the twelve subregions (ICC = 0.66–0.96); however, the hippocampal fissure was not consistently reconstructed across 1.5 T and 3 T field strengths (ICC = 0.47–0.57). Between-version agreement was moderate for the hippocampal tail, subiculum and presubiculum (ICC = 0.78–0.84; Dice Similarity Coefficient (DSC) = 0.55–0.70), and poor for all other subregions (ICC = 0.34–0.81; DSC = 0.28–0.51). All hippocampal subregion volumes were highly heritable (*h*^2^ = 0.67–0.91). Our findings indicate that eleven of the twelve human hippocampal subregions segmented using *FreeSurfer* version 6.0 may serve as reliable and informative quantitative phenotypes for future multi-site imaging genetics initiatives such as those of the ENIGMA consortium.

## Introduction

The mammalian hippocampal formation is one of the most important brain regions for spatial navigation (O’Keefe, 1990), episodic memory retrieval (Burgess et al., 2002), and associative learning processes (Morris, 2006). This seahorse-shaped structure in the medial temporal lobe is divided into a set of cytoarchitectonically heterogeneous subregions (Insausti and Amaral, 2004; Winterburn et al., 2013; Pipitone et al., 2014), each associated with distinct aspects of memory formation, among other functions. For example, the dentate gyrus (DG) and sectors 3 and 4 of the *cornu ammonis* (CA) are involved in declarative memory acquisition (Coras et al., 2014), whereas the subiculum and CA1 are associated with disambiguation during working memory processes(Newmark et al., 2013).The CA2 subregion, long assumed to be a simple transition point between CA3 and CA1, has recently been implicated in animal models of social memory (Hitti and Siegelbaum, 2014) and episodic time encoding (Navratilova and Battaglia, 2015). The subiculum, a subregion that exerts control over the hippocampal output, has been associated with spatial memory functions, but its ventral part may play an additional regulatory role in inhibition of the HPA axis (O’Mara, 2006).

Neuroanatomical abnormalities within these hippocampal subregions are associated with a broad range of neurological and psychiatric disorders, from ischaemic stroke, encephalitis, temporal lobe epilepsy, transient global amnesia and multiple sclerosis (Bartsch, 2012; Das et al., 2011) to bipolar disorder (BPD), major depressive disorder (MDD) and posttraumatic stress disorder (PTSD) (Sala, 2008). Some of these malformations develop as a result of head trauma, intracranial infection or other environmental influences, but genetic factors also play a fundamental role (Thompson et al., 2008; van Erp et al., 2004). Recent advances in genome-wide association (GWA) meta-analysis and large-scale collaborative brain imaging (*e.g*. Enhancing Neuro Imaging Genetics through Meta-Analysis (ENIGMA), the Early Growth Genetics (EGG) consortium, and the Cohorts of Heart and Aging Research in Genomic Epidemiology (CHARGE)) have helped identify several common genetic variants associated with structural variation in the hippocampus (Bis et al., 2012; Hibar et al., 2015; Stein et al., 2012) as well as other brain regions including the putamen, caudate nucleus (Hibar et al., 2015), intracranial volume (Ikram et al., 2012; Stein et al., 2012) and head circumference (Taal et al., 2012).

International consortia like ENIGMA are now turning their attention to specific investigations of genetic and phenotypic variation in healthy individuals as well as those diagnosed with schizophrenia, BPD, MDD, PTSD, epilepsy and many other brain illnesses (Thompson et al., 2014). Among subcortical structures assessed, the hippocampus has consistently shown the greatest effect sizes for differences between patients and controls, in both schizophrenia (van Erp et al., 2015) and major depression, particularly recurrent depression (Schmaal et al., 2015). Impaired hippocampal integrity may in turn impair treatment response, making it pivotal to detect such morphologically defined subgroups (Frodl et al., 2008; Sämann et al., 2013).

Focusing on fine-grained phenotypic variation within small subregions of the hippocampus may improve our power to localize genetic and disease-related effects on the brain as a whole. As part of its next major project, the ENIGMA consortium aims to delineate specific sub-regions of the hippocampus as quantitative phenotypes for genome-wide association and cross-sectional case:control meta-analyses. Before these new ENIGMA initiatives can begin, we first need to evaluate a non-invasive, reliable and relatively accessible technique for reconstructing the human hippocampal subfields *in vivo*. In turn, for future genetic mapping efforts, we must validate these automatically reconstructed hippocampal sub-regions as quantitative *endophenotypes* — heritable, robust brain markers that may be closer to the molecular basis of disease than diagnostic assessments in the clinic (Braskie and Ringman, 2011; Glahn et al., 2007; Gottesman and Gould, 2003; Hasler and Northoff, 2011).

Several manual segmentation techniques have been developed to reconstruct hippocampal and parahippocampal subregions from T1-weighted MRI scans acquired at 3 to 7 T field strengths (Adler et al., 2014; La Joie et al., 2010; Van Leemput et al., 2009; Mueller et al., 2007; Wisse et al., 2012). Although these methods typically segment the hippocampal subregions at remarkably fine-scaled resolution, a critical bottleneck for collaborative imaging initiatives such as ENIGMA is the need to manually label the subregion boundaries, which is laborious, time-consuming and susceptible to intra- and inter-observer variability (Van Leemput et al., 2009). Several automated protocols have been developed to address this issue, combining rules on image intensity and geometry to delineate the boundaries between hippocampal and parahippocampal subregions (Van Leemput et al., 2009; Yushkevich et al., 2009, 2010). One often-used automated technique is provided as part of FreeSurfer, a freely available suite of neuroimaging structural analysis tools (Fischl, 2012).

Initial versions of the FreeSurfer algorithm (versions 5.1,5.2 and 5.3) produce subregion segmentations that are largely inconsistent with brain anatomy (de Flores et al., 2015; Pluta et al., 2012; Wisse et al., 2014). An updated version of the algorithm, to be released as part of FreeSurfer version 6.0, uses a new statistical atlas constructed from ultra-high resolution *ex vivo* MRI (Iglesias et al., 2015). This revised algorithm produces subregion volume estimates that more closely match volumes derived from histological investigations (Iglesias et al., 2015). However, consensus is still lacking on the most appropriate subregion delineation protocol to use (Yushkevich et al., 2015). Here, using four independent samples, we set out to validate version 6.0 of the automated FreeSurfer algorithm from three complementary perspectives: First, we evaluated the algorithm’s ‘test-retest’ reliability; *i.e.* its ability to extract comparable subregion measures across multiple time points in two independent cohorts with different image acquisition parameters and age characteristics (our two samples differ in mean age by approximately 50 years). Second, we examined the algorithm’s ‘trans-platform’ reliability — defined as its ability to reproduce similar subregion measures across different MRI scanner platforms and field strengths (for example, 3 T *versus* 1.5 T). Third, we investigated overall agreement between this new algorithm, which we will refer to as ‘FS6.0’, and the older algorithm, version 5.3, which we will refer to as ‘FS5.3’. The degree of quantitative deviation between volumes extracted using FS5.3 and volumes extracted using FS6.0 may help users of the former evaluate the necessity of re-processing their data with the latter.

Validation of a reliable, automated subregion segmentation tool may allow ENIGMA and other imaging consortia to study hippocampal subregions as fine-grained quantitative phenotypes in large-scale genome-wide association meta-analyses. However, to be considered a promising target for genetic mapping, the subregional volume estimates must show evidence of heritability (*h*^2^). Quantitative genetic analysis of automatically segmented, T1-weighted brain images from paired twin samples has frequently been employed to estimate the heritability of global volumetric measures. Prior estimates show that total hippocampal volume is highly heritable in both healthy adults (*h*^2^ = 0.66–0.71) (den Braber et al., 2013; van Erp et al., 2004; Wright et al., 2002) and children (*h*^2^ = 0.64–0.72) (Swagerman and Brouwer, 2014). However, structural variance within the whole hippocampus may be less heritable in elderly adults (*h*^2^ = 0.4–0.65) (DeStefano et al., 2009; Mather et al., 2015; Sullivan et al., 2001), possibly due to environmental stressors (Hedges and Woon, 2010), alterations in testosterone levels (Panizzon et al., 2012) or other endogenous biological factors. Similarly, total hippocampal volume is only moderately heritable in schizophrenia (*h*^2^ = 0.36–0.73) (Kaymaz and Os, 2009; Roalf et al., 2015). Thus, while the heritability of total hippocampal volume is well established across many populations, the heritability of structural variations in individual subregions has yet to be delineated. Therefore, in the second part of this study, we set out to disentangle the relative contributions of additive genetic variance and environmental influences on hippocampal subregion volume in two independent cohorts of healthy adults, and by this to assess the eligibility of such hippocampal subregion volumes as *endophenotypes* for future large-scale collaborative genetic association studies in ENIGMA.

## Methods

### Participants and imaging protocols

Four collections of MRI scans were analyzed in this study.

### ADNI-2

#### Subjects

For our test–retest and between-version reliability analyses, we analyzed publicly available data from 163 healthy control subjects from the second phase of the Alzheimer’s Disease Neuroimaging Initiative, ADNI-2 (81 women, 82 men, age mean ± SD = 73.58 ± 6.21 years) (http://adni.loni.usc.edu/). ADNI was launched in 2003 as a public-private partnership, led by Principal Investigator Michael W. Weiner, MD. The primary goal of ADNI has been to test whether serial magnetic resonance imaging (MRI), positron emission tomography (PET), other biological markers, and clinical and neuropsychological assessment can be combined to measure the progression of mild cognitive impairment (MCI) and early Alzheimer’s disease (AD). Further details of the ADNI project are given in Jack et al. (2010) and at http://www.adni-info.org.

#### Imaging

T1-weighted MR images were acquired using a 3 T General Electric (GE) Medical Systems scanner with the following parameters: 3-dimensional MP-RAGE, 8-channel head coil, voxel size 1.2 × 1.2 × 1.2 mm, time to repeat (TR) = 400 ms, time to echo (TE) = 2.85 ms, flip angle = 11°, field of view (FOV) = 26 cm, resolution = 256 × 256 mm. A baseline and follow-up scan was acquired for all healthy controls, with an average inter-scan interval of 3.3 months. Family trios or siblings were not scanned as part of the ADNI-2 protocol, so this dataset was not included in our heritability analyses.

### QTIM

#### Subjects

To estimate heritability and include an independent replication cohort for our test–retest reliability analysis, we analyzed MR images from healthy Caucasian young adults, collected as part of the Queensland Twins Imaging (QTIM) study. QTIM is a joint effort by researchers at QIMR Berghofer, The University of Queensland and the University of Southern California to study brain structure and function using T1-weighted MRI, high angular resolution diffusion imaging (HARDI) and functional MRI in a large population of young adult twins of European ancestry. Full details of the QTIM cohort are found in Zubicaray et al. (2008).

The heritability analysis included 728 individuals (132 monozygotic (MZ) sibling pairs and 232 dizygotic (DZ) sibling pairs; 465 women and 263 men with an age mean ±SDof 22.65± 2.73 years). The test–retest reliability analysis included a subset of the twins; 20 women, 19 men; mean age in years (±SD) = 24.03 (±2.04), who were scanned twice, with an average interval of 3 months between scanning sessions.

#### Imaging

3-Dimensional T1-weighted images were acquired on a 4 T Bruker Medspec scanner using an inversion recovery rapid gradient echo protocol. Key acquisition parameters were: TI = 700 ms, TR = 1500 ms, TE = 3.35 ms, voxel size 0.94 × 0.98 × 0.98 mm, flip angle = 8°, slice thickness = 0.9 mm, 256 × 256 acquisition matrix.

### Max Planck Institute of Psychiatry (MPIP)

#### Subjects

As part of the (i) between-version agreement and (ii) transplatform reliability analyses, high resolution T1-weighted anatomical images collected at the Max Planck Institute of Psychiatry (MPIP), Munich, Germany, from 222 healthy participants and 367 patients with major depressive disorder (MDD) (334 women, 255 men, mean age ± SD = 48.4 ± 13.5, age range: 18 to 87), were included, in addition to 20 healthy controls who were scanned on a 1.5 T and 3 T platform.

#### Imaging

The between-version comparison sample (total N = 589) was acquired on a 1.5 T General Electric clinical scanner (T1-weighted SPGR 3D volume, TR 10030 ms; TE 3.4 ms; 124 sagittal slices; matrix 256 × 256; FOV 23.0 × 23.0 cm^2^; voxel size 0.8975 × 0.8975 × 1.2– 1.4] mm^3^; flip angle = 90°; birdcage resonator) with N = 186 of the total sample scanned after a coil upgrade (Signa Excite, sagittal T1-weighted spin echo sequence, TR 9.7 s, TE 2.1 ms). For the trans-platform sample, one image was acquired on 3 T scanner (General Electric MR750, 3D BRAVO, TR 6.1 s; TE minimum; TI 450 ms, 124 sagittal slices; matrix 256 × 256; FOV 25.6 × 25.6 cm^2^; voxel size 1×1×1 mm^3^; flip angle = 12°) and a second image after immediate repositioning in the 1.5 T scanner (General Electric MR450, 3D FSPGR, TR 7.9 s; TE minimum, TI 450 ms, 188 sagittal slices; matrix 320 × 256; FOV 24 × 24 cm^2^; voxel size 0.9375 × 0.9375 × 1 mm^3^; flip angle = 12°).

### Netherlands Study of Depression and Anxiety (NESDA)

#### Subjects

To further assess the agreement between FreeSurfer versions, we analyzed data from 64 healthy controls and 157 patients with a diagnosis of MDD or comorbid anxiety disorder, collected as part of the Netherlands Study for Depression and Anxiety (NESDA) (145 women, 76 men, mean age ± SD = 38.14 ± 10.33 years, age range: 18 to 57).

#### Imaging

Imaging data were acquired using Philips 3 T magnetic resonance imaging systems (Best, The Netherlands) located at the Leiden University Medical Center, Amsterdam Medical Center, and University Medical Center Groningen. For each subject, anatomical images were obtained using a sagittal 3-dimensional gradient-echo T1-weighted sequence (repetition time, 9 ms, echo time, 3.5 ms; matrix, 256 × 256; voxel size, 1×1×1 mm; 170 slices; duration, 4.5 min).

Full participant demographics for the ADNI-2, QTIM, MPIP and NESDA samples are detailed in Table 1.

### Image processing

T1-weighted images were processed using FreeSurfer (FS) version 5.3.0 using the software package’s default, automated reconstruction protocol described by Anders M. Dale, Bruce Fischl and colleagues (‘recon-all’–see Dale et al., 1999; Fischl et al., 1999). Briefly, each T1-weighted image was subjected to an automated segmentation process involving: (i) conversion from three-dimensional *nifti* format, (ii) affine registration into Talairach space, (iii) normalization for variable intensities caused by inhomogeneities in the radiofrequency field, (iv) ‘skull-stripping’, *i.e.* extraction of the skull and extrameningeal tissues from each image, (v) segregation into left and right hemispheres using ‘cutting planes’, (vi) removal of the brain stem and cerebellum, (vii) correction for topology defects, (viii) definition of the gray/white matter and gray/cerebrospinal fluid boundaries using surface deformation (Fischl et al., 2004a) and (ix) parcellation of the subcortical region into distinct brain tissues, including the hippocampus, amygdala, thalamus, caudate nucleus, putamen, pallidum and accumbens (Fischl et al., 2002, 2004a, 2004b). Using FreeSurfer’s native visualization toolbox, *tkmedit*, we visually inspected each image for over- or under-estimation of the gray/white matter boundaries and to identify brain areas erroneously excluded during skull stripping.

### Hippocampal subregion segmentation

After successful reconstruction of the whole hippocampus and its neighboring subcortical regions, we used a revised version of the automated subregion parcellation protocol previously described by Van Leemput and colleagues (Van Leemput et al., 2009) to segment specific subregions of the hippocampal formation in the QTIM, ADNI-2, NESDA and MPIP datasets. This revised module is compatible with FreeSurfer v5.3 (FS5.3) and will be freely distributed with FreeSurfer v6.0 (FS6.0) (Iglesias et al., 2015). Prior versions of the algorithm (FS5.1 to FS5.3) combined a single probabilistic atlas with high-resolution, T1-weighted *in-vivo* manual segmentations to predict the locations of eight hippocampal subregions. The new version (FS6.0) predicts the location of twelve hippocampal subregions, using a refined probabilistic atlas built upon a combination of manual delineations of the hippocampal formation from 15 ultra-high resolution, *ex-vivo* MRI scans and manual annotations of the surrounding subcortical structures (*e.g.*, amygdala, cortex) from an independent dataset of 39 *in-vivo*, T1-weighted, 1 mm resolution MRI scans (Iglesias et al., 2015). This revised algorithm features the following enhancements: (i) first-hand knowledge of histological staining of the hippocampus by a neuroanatomist; (ii) a cytoarchitectural atlas of the hippocampal formation (Rosene and Hoesen, 1987); and (iii) highresolution, *ex-vivo* brain MRI scans (120 μm^3^), which show definitive borders between the subregions and greater consistency with manual segmentation methods (Yushkevich et al., 2015). Previous versions of the FreeSurfer algorithm reconstructed eight subregions per hemisphere, including the CA1, CA2/3, fimbria, subiculum, presubiculum, CA4/DG, hippocampal tail and hippocampal fissure. The new algorithm provides more anatomically sensitive reconstructions of these eight subregions as well as four new subregions: the parasubiculum, the molecular layer, granule cells in the molecular layer of the DG (GC-ML-DG) and the hippocampal-amygdala transitional area (HATA).

### Test–retest reliability analysis

**Fig. 1:**
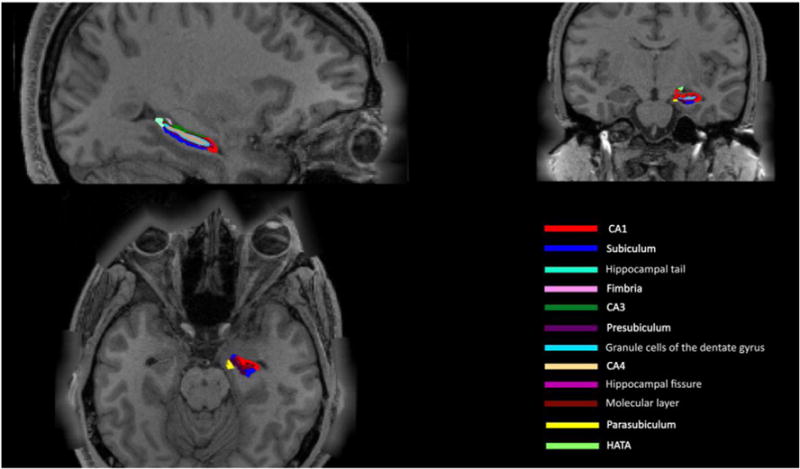
Color-coded illustration of 11 hippocampal subfields in sagittal (top left), axial (bottom left) and coronal (top right) views. Subfield volumes for each participant were overlaid on their whole-brain T1-weighted image (‘nu.mgz’) and visually inspected for over- or under-estimation of the hippocampal subfields. In the above rendering, a representative subject from the QTIM cohort was de-identified by blurring around the edges of the skull and face. The image was generated using FreeSurfer’s high-resolution visualization tool, *FreeView* (https://surfer.nmr.mgh.harvard.edu/fswiki/FreeviewGuide/).

Using FS6.0, we extracted volume estimates for the whole hippocampus and its twelve subregions from (i) the ADNI-2 and (ii) the QTIM cohorts. All QTIM and ADNI-2 images, including both test and re-test scans, were processed in parallel. After successful subregion segmentation, we used a custom-designed Matlab code to visually inspect each segmentation (see Fig. 1). Subregion volume estimates were exported to SPSS (for reliability analysis) and reformatted into phenotype covariance matrices (for heritability analysis described below).

Volume measures were imported into SPSS (IBM Corp., Version 21.0) and subjected to a series of two-way reliability analyses, using Cronbach’s alpha (α) (Cronbach, 1951) as a measure of internal consistency. Cronbach’s alpha is calculated as follows:

$$\propto =\frac{N\cdot \bar{c}}{\bar{\bar{v}+(N-1)\cdot \bar{c}}}$$

where *N* is the number of subregion volume estimates, *c-bar* is the average inter-subject covariance among these estimates and *v-bar* is the average variance. The resulting α, interpreted as the *intraclass correlation coefficient* (ICC), provides an estimate of how consistently the FreeSurfer v6.0 parcellation protocol reconstructs hippocampal subregions from baseline to follow-up scan. ICC ranges from 0 (indicating high variability between baseline and follow-up volume estimates) to 1 (denoting high reproducibility between baseline and follow-up estimates).

### Between-version reliability analysis

We compared subregional hippocampal volumes estimates extracted using FS5.3 and FS6.0 from three independently acquired cohorts: (i) baseline scans of the ADNI-2 cohort (N = 163), (ii) the NESDA cohort (N = 221), and (iii) the MPIP cohort (N = 589). Volume measures for each subregion were bilaterally ‘averaged’ across the left and right hemispheres.

Volume measurements from FS6.0 are given in mm^3^, whereas volume measurements in FS5.3 are returned on the basis of 0.5 mm isotropic. Therefore, the latter set of volume estimates was divided by a factor of 8 in order to transform them to mm^3^ measurements.

Volume estimates for the eight sub-regions extracted using FS5.3 were imported into SPSS alongside eight of the twelve possible subregions extracted using FS6.0. Volume estimates for the parasubiculum, molecular layer, GC_ML_DG and HATA (extracted using FS6.0) had no direct corresponding subregions in FS5.3 and were not included in this between-version analysis. We conducted eight sets of two-way mixed reliability analyses, using the same statistical model applied for our prior test–retest comparison (Cronbach’s alpha). This produced a series of ICC values measuring the agreement between the old (FS5.3) and new (FS6.0) versions of the FreeSurfer subregion segmentation algorithm.

As a second measure of reproducibility and spatial overlap between FS5.3 and FS6.0, we employed a custom-designed Matlab code to extract a series of Dice similarity coefficients (DSC) for each hippocampal subregion. The DSC, first proposed by Dice (1945), provides a validation metric for evaluating reproducibility and has previously been used to assess spatial overlap between automated MRI reconstructions (Zou et al., 2006). DSC values range from 0 (indicating no spatial overlap between two sets of binary segmentations) to 1 (full overlap between binary segmentations).

DSCs were calculated by dividing the sum of volumes segmented using FS5.3 and volumes segmented using FS6.0 by twice the volume of the intersection between these segmentations; *i.e.*

DSC(A,B)=2(A∩B)/(A+B)

where *A* is the first hippocampal subregion (reconstructed using FS5.3), *B* is the second hippocampal subregion volume (reconstructed using FS6.0) and ∩ is the intersected space between the two subregions.

### Trans-platform reliability analysis

20 pairs of T1-weighted images were acquired on a 1.5 T and a 3 T scanner system to investigate the stability of both FS5.3 and FS6.0 across platforms. The repositioning between the end of the first acquisition and the start of the second acquisition was performed as fast as possible, usually taking 2–3 min. Both subregional segmentation tools (FS5.3 and FS6.0) were employed on the 2 × 20 images. Subregional volume estimates were imported into SPSS (to extract ICC values) and Matlab (to estimate DSC scores) respectively. All ICC analyses were conducted using the same statistical models previously described for the test–retest analysis.

### Heritability of hippocampal subregion volumes

Heritability, defined here as the fraction of the phenotypic variability attributable to genetic variation, was calculated for each hippocampal subregion volume using a variance components model, as implemented in version 7.2.5 of the Sequential Oligogenic Linkage Analysis Routines (SOLAR) software package (http://www.nitrc.org/projects/se_linux) (Almasy and Blangero, 1998). Methods to estimate heritability in SOLAR are detailed elsewhere (Kochunov et al., 2010; Winkler et al., 2010).

Briefly, SOLAR implements a maximum likelihood variance decomposition method, expanding on prior algorithms developed by Amos (1994). The algorithm decomposes phenotypic variance (σ^2^_P_) into a genetic $\left(\sigma_{g}^{2}\right)$ and a residual component $\left(\sigma_{e}^{2}\right)$ — the latter represents variation not accounted for by the genetic component (*i.e.*, random environmental variation and/or experimental error). Mean volumes for the whole hippocampus and twelve of its subregions were extracted from all twin pairs in the QTIM sample (N = 132 MZ pairs and N = 232 dizygotic pairs) and reformatted into a phenotype covariance matrix. Each covariate matrix was adjusted to include sex, age, and age * sex interactions as covariates. The covariance matrix, Ω, for each pedigree of individuals was then integrated into the following expression:

$$\Omega =2\Phi {\sigma_{g}}^{2}+I{\sigma_{e}}^{2}$$

where Ω represents covariance between one relative and another, Φ is the pair-wise kinship coefficient representing the relationship between these relatives (0.5 for full siblings), $\left(\sigma_{g}^{2}\right)$ represents the additive genetic component of phenotypic variance, *I* is the identity matrix and $\left(\sigma_{e}^{2}\right)$ is residual non-genetic variation (i.e., individual-specific environmental variance).

Heritability (*h*^2^) was computed from this model by comparing the observed covariance matrix for phenotypic variance $\left(\sigma_{p}^{2}\right)$ with the observed covariance matrix for additive genetic effects $\left(\sigma_{g}^{2}\right)$, i.e.,

$$h^{2}={\sigma^{2}}_{g}/{\sigma^{2}}_{p}.$$

Here, *h*^2^ is a value between 0 and 1 representing total additive genetic heritability, ranging from 0 (no genetic contributions) to 1 (all phenotypic variance reflects a genetic effect). Significance of heritability was estimated by computing a model in which σ^2^_g_ was constrained to zero, computing a second model in which σ^2^_g_ was estimated, and computing twice the difference between the first and second models’ log-likelihoods. For our analysis, we employed a polygenic model that calculated the effects of specific variables (additive genetic variation, and covariates including age, sex and sex * age interactions) in explaining each subregion’s volumetric variance within the QTIM population. Three main test statistics were then recorded for each subregion volume: its *h*^2^ estimate, the significance (*p*-value) of this heritability estimate and its standard error. All test statistics were compared to an adjusted alpha level of *p* **≤** 3.84 × 10^−3^ to reduce the probability of type 1 errors arising from multiple measurements (N = 13).

## Results

### Test–retest reliability

Test–retest reliability estimates from ADNI-2, a cohort of 163 healthy, elderly adults scanned three months apart at 3 T, revealed good reliability for all automatically segmented subregion volumes. Larger hippocampal regions (mean volume > 90 mm^3^) showed highest ICC values from baseline to follow-up session. These regions included the whole hippocampus (ICC ≥ 0.94), CA1 subregion (ICC ≥ 0.91), CA3 subregion (ICC ≥ 0.88), CA4 subregion (ICC ≥ 0.9), molecular layer (ICC ≥ 0.93), subiculum (ICC ≥ 0.91), presubiculum (ICC ≥ 0.9), granule cells (ICC ≥ 0.91), hippocampal tail (ICC ≥ 0.93), hippocampal fissure (ICC ≥ 0.88) and fimbria (ICC ≥ 0.89). Automated segmentation was also stable for smaller subregions, including the HATA (ICC ≥ 0.78) and parasubiculum (ICC ≥ 0.75) (see Table 2).

Similarly, in the smaller QTIM sub-sample, consisting of 39 young, healthy adults scanned on average three months apart at 4 T, we found strong test–retest reliability for large subregions (mean volume > 90 mm^3^). These subregions included the CA1 (ICC ≥ 0.86), CA3 (ICC ≥ 0.78), CA4 (ICC ≥ 0.75), molecular layer (ICC ≥ 0.86), subiculum (ICC ≥ 0.8), granule cells (ICC ≥ 0.78), hippocampal tail (ICC ≥ 0.72), hippocampal fissure (ICC ≥ 0.7) and fimbria (ICC ≥ 0.8), as well as the whole hippocampus (ICC ≥ 0.85). Test–retest reliability of the presubiculum varied considerably from the left (ICC = 0.89) to the right hemisphere (ICC = 0.65). Volume estimates were moderately reproduced for the parasubiculum (ICC ≥ 0.68) and the HATA subregion (ICC ≥ 0.5).

### Between-version agreement

In the MPIP cohort (N = 589, 3 T) we found strong agreement between versions 5.3 and 6.0 of the FreeSurfer segmentation algorithm for the subiculum (0.857). We observed moderate agreement between the following subregions: (i) the hippocampal tail (ICC = 0.778), (ii) the fimbria (ICC = 0.78), (iii) the hippocampal fissure (ICC = 0.78) and (iv) the presubiculum (ICC = 0.797). Agreement between the three major sectors of the *cornu ammonis* (CA1, CA2_3 and CA4) varied considerably; for example, the CA1 (extracted using FS6.0) showed strong agreement with the CA4/Dentate (extracted using FS5.3; ICC = 0.872) and CA2_3 (extracted using FS5.3; ICC = 0.817) but only moderately correlated with its direct counterpart, CA1 (extracted using FS5.3; ICC = 0.645). Similarly, the CA4 subregion extracted using FS6.0 only moderately correlated with the combined CA4-DG from FS5.3 (ICC = 0.66), whereas the CA3 extracted using FS6.0 correlated poorly with its closest counterpart in FS5.3, the CA2_3 (ICC = 0.383) (see Table 3).

The second set of ICCs, examining between-version agreement using volume estimates from the ADNI-2 cohort (N = 163, 3 T), revealed strong agreement between versions 5.3 and 6.0 for (i) the hippocampal tail (ICC = 0.839), (ii) the fimbria (ICC = 0.805), (iii) the presubiculum (ICC = 0.825) and (iv) the subiculum (ICC = 0.833). Between-version agreement was moderate for the hippocampal fissure (ICC = 0.628) and the CA4 (ICC = 0.633). The CA1 subregion (segmented using FS6.0) showed greater correspondence with FS5.3 reconstructions of the CA4_DG (ICC = 0.872) and CA2_3 (ICC = 0.817) than its direct anatomical counterpart, the CA1 (ICC = 0.645). Similarly, the CA3 (showed poor correlation between FS5.3 and FS6.0 (ICC = 0.344), although correlations were higher between the CA3 (extracted using FS6.0) and other subregions from FS5.3, including the CA1 (ICC = 0.523) and CA4_DG (ICC = 0.567) (see Table 4).

The third set of ICCs examined between-version agreement using values extracted from the NESDA cohort (N = 221, 3 T). This analysis revealed strong agreement between FS5.3 and FS6.0 for the subiculum (ICC = 0.815) and moderate agreement for the following subregions: (i) hippocampal tail (ICC = 0.778), (ii) fimbria (ICC = 0.758) and (iii) presubiculum (ICC = 0.783). CA1 volumes extracted using FS6.0 correlated moderately with CA1 volumes extracted using FS5.3 (ICC = 0.698), but correlated more highly with CA4_DG volumes extracted using FS5.3 (ICC = 0.856). Similarly, CA4 volumes extracted using FS6.0 correlated moderately with CA4_DG volumes from FS5.3 (ICC = 0.592), but correlated more highly with CA1 volumes from FS5.3 (ICC = 0.729). Further, the CA3 subregion extracting using FS6.0 correlated poorly with the CA2_3 subregion extracted using FS5.3 (ICC = 0.334), but correlated moderately with the CA1 (0.679) and CA4_DG (0.545). Between-version agreement was poor for the hippocampal fissure (ICC = 0.321) (see Table 5).

A complementary analysis of spatial overlap and reproducibility (as measured by the Dice Similarity Coefficient, DSC) revealed high spatial overlap across the ADNI-2, MPIP and NESDA cohorts for the whole hippocampus (DSC = 0.82–0.85). Between-version agreement was moderate for the hippocampal tail across the three cohorts (DSC = 0.67–0.70). Between-version agreement was poor-to-moderate for the CA4_DG (DSC = 0.49–0.51), fimbria (DSC = 0.45–0.53), presubiculum (DSC = 0.57–0.62) and subiculum (DSC = 0.55–0.58). Between-version agreement was poor for the CA1 (DSC = 0.39–0.4) and the CA2_3 (DSC = 0.28–0.30; see Table 6).

### Trans-platform reliability

We conducted two sets of intraclass correlations, testing reliability across two MRI scanner platforms – 1.5 T and 3 T – using (i) FS5.3 and (ii) FS6.0, respectively. The subregion segmentation algorithm provided as part of FS5.3 produced stable volume estimates across scanning platforms for the following regions: (i) the whole hippocampus (ICC = 0.855), (ii) the CA2_3 (ICC = 0.856), (iii) the CA4/dentate (ICC = 0.892), (iv) the presubiculum (ICC = 0.818), (v) the subiculum (ICC = 0.866), (vi) the hippocampal tail (ICC = 0.875), (vii) the CA1 (ICC = 0.725) and (iix) the fimbria (ICC = 0.720). Volume estimates were not reliably reproduced across scanner platforms for the hippocampal fissure (ICC = 0.465) (see Table 7).

The subregion segmentation algorithm provided as part of FS6.0 produced high ICC estimates for the following regions: (i) the whole hippocampus (ICC = 0.942), (ii) the subiculum (ICC = 0.858), (iii) the CA1 (ICC = 0.915), (iv) the presubiculum (ICC = 0.853), (v) the molecular layer (ICC = 0.932), (vi) the granule cells of the dentate gyrus (ICC = 0.932), (vii) the hippocampal tail (ICC = 0.863), (iix) the CA3 (ICC = 0.827), (ix) the HATA (ICC = 0.801), (x) the CA4 (ICC = 0.792) and (xi) the fimbria (ICC = 0.721). Volume estimates were moderately correlated between scanning platforms for the parasubiculum (ICC = 0.659) and the hippocampal fissure (ICC = 0.575) (see Table 7).

### Heritability of hippocampal subregion volumes

**Fig. 2:**
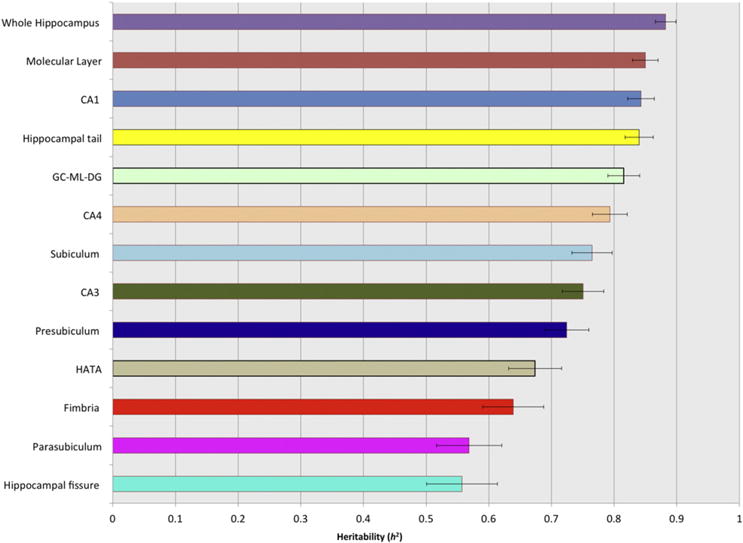
Heritability of the whole hippocampus and its respective subfields in the QTIM cohort (N = 728).

**Fig. 3:**
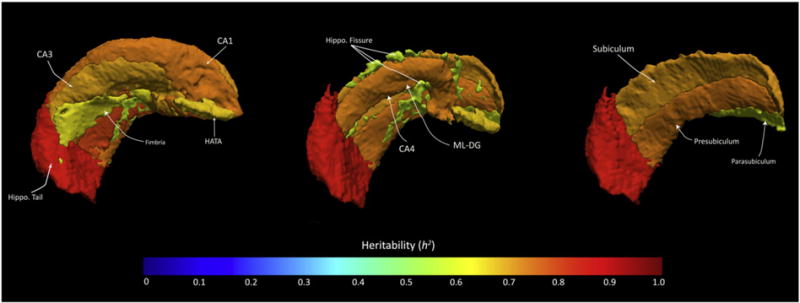
Three-dimensional visualization of narrow-sense heritability within twelve subfields of the human hippocampal formation, using the average heritability estimates calculated from the QTIM cohort. Heritability is represented as a heat map, with the most heritable subregions depicted in red (see: the hippocampal tail) and moderately heritable subfields colored in green/yellow (see: the hippocampal fissure and parasubiculum). The first image (on the left) is a full reconstruction of the hippocampal formation, showing the most lateral subfields including the CA1, CA3, hippocampal tail (‘*hippo. tail*’), fimbria and hippocampal-amygdaloid transition area (‘*HATA*’). The middle image removes some lateral substructures, including the fimbria and CA3, in order to display mid-lying subfields including the hippocampal fissure (‘*hippo. fissure*’), molecular layer and granule cells of the DG (‘*ML-DG*’) and CA4. The third image (on the right) further removes these subfields in order to display three remaining medial sub-regions, including the subiculum, presubiculum and parasubiculum. This rendering represents bilateral h^2^ estimates, although only the left hippocampus is shown here. Image generated using TrackVis (http://trackvis.org/).

Fig. 2 shows the proportion of structural variance attributable to genetic factors for the whole hippocampus and its subregions in the QTIM sample. All regions exhibited high heritability, between 0.56 and 0.88. The highest heritability estimates (*h*^2^ ≥ 0.7) were observed for large regions with mean volumes of 220 mm^3^ or greater (i.e., the whole hippocampus, molecular layer, CA1, CA3, CA4, hippocampal tail, granule cell layer, subiculum and presubiculum). Smaller subregions (mean volume: 60–165 mm^3^) showed moderate-to-high heritability (0.55 < h^2^ < 0.7) (see Fig. 2). Table 8 shows the heritability estimates alongside their significance values and standard errors. Using a combination of FreeSurfer subregion labels and TrackVis (http://trackvis.org/), we constructed a three-dimensional visualization of each heritability estimate, this shows how large, posterior subregions (i.e., the hippocampal tail) were most heritable, whereas smaller, anteromedial subregions (parasubiculum, presubiculum and fimbria) were less influenced by genetic factors (see Fig. 3).

## Discussion

Here we evaluated a series of automatically segmented volumetric measures from the hippocampus and twelve of its major subregions as reliable, heritable quantitative phenotypes for future large-scale imaging genetics studies. We had four main findings. First, the most recent version of a widely employed FreeSurfer segmentation protocol (FS6.0) showed good test–retest reliability, both at3 T and 4 T in healthy young and older adults. Spatial overlap between segmentations produced at baseline and follow-up time points was moderate-to-high for all subregions, with the exception of the hippocampal fissure. Second, segmentations produced using FreeSurfer v6.0 showed strong reproducibility from 1.5 T to 3 T field strengths. Third, subregional volume estimates varied between prior and revised versions of the FreeSurfer algorithm, with some subregions (e.g. the hippocampal tail) remaining stable, and others (*e.g.* the *cornu ammonis*) diverging notably from one version to the next. Fourth, genetic factors significantly affected the volume of the human hippocampus and its twelve major subregions in a sample of healthy, adult twins. Multi-site genetic analysis may therefore be feasible for automatically extracted subregion measures, building on prior studies that detected common variants associated with overall hippocampal volume (Stein et al., 2012; Hibar et al., 2015).

### FreeSurfer v6.0: Reliable test–retest segmentations of eleven hippocampal subregions

Automated parcellation algorithms are essential neuroimaging tools, as they facilitate the harmonized, time-efficient and precise reconstruction of brain regions across multiple sites. The automated subcortical segmentation protocol included in the *FreeSurfer* software package has been employed in several important imaging collaborations, leading to the discovery of genetic polymorphisms associated with subcortical and intracranial volumes (Hibar et al., 2015; Ikram et al., 2012; Stein et al., 2012) and the identification of robust subcortical alterations in large populations of people with schizophrenia (Van Erp et al., 2015) and major depressive disorder (Schmaal et al., 2015). FreeSurfer has been validated as a reliable method to reconstruct and measure larger brain regions (Jovicich et al., 2006; Wonderlick et al., 2009), but early versions of its hippocampal subregion segmentation module were criticized by some as anatomically inaccurate, overly reliant on low-resolution images and not yet validated against manual tracing techniques (de Flores et al., 2015; Pluta et al., 2012; Wisse et al., 2014). Here, we found that a revised version of the FreeSurfer subregion segmentation tool, due to be released with FreeSurfer v6.0, produces reliable segmentations for eleven of the twelve hippocampal subregions at 3 T and 4 T field strengths. The most reliably reconstructed sub-regions included the hippocampal tail, CA1, CA4, presubiculum and subiculum. These subregions showed excellent test–retest reliability in two independent tests (ICC and DSC analysis) and in two unrelated cohorts (ADNI and QTIM).

Other subregions, including the dentate gyrus, CA3, fimbria, HATA and parasubiculum, showed strong test–retest reproducibility at 3 T field strength, but a wider range of test–retest reproducibility at 4 T field strength. This discrepancy may be explained, in part, by the smaller sample size of the 4 T cohort (QTIM; N = 39) compared to the 3 T cohort (ADNI-2; N = 163). ICC estimates extracted from the 4 T cohort were associated with larger confidence intervals (CIs), many of which overlapped with CIs from the 3 T cohort (see Table 2). Voxel size differences between ADNI-2 (1.2 × 1.2 × 1.2 mm) and QTIM (0.94 × 0.98 × 0.98 mm) may have also contributed towards these discrepancies: FreeSurfer resamples MR images to 1 mm isotropic voxel size during its automated reconstruction process and this interpolation procedure may produce variable resolutions in datasets that are ‘down-sampled’ (*i.e.* ADNI-2) compared to those that are ‘up-sampled’ (*i.e.* QTIM).

Of the twelve subregions we investigated, only one – the hippocampal fissure – produced unreliable volume estimates between baseline and follow-up acquisitions. The hippocampal fissure is a vestigial sulcus located between the molecular layer of the hippocampus and the dentate gyrus. Several neuroanatomical and methodological variables may contribute to the inconsistent segmentation of this subregion. Its relatively small size and complex cytoarchitectural morphometry may make the subregion more susceptible to partial volume effects caused by changes in the subject’s head positioning, variable tissue contrast profiles or even small, undetected changes in the MR signal (Morey et al., 2010). The relatively arbitrary boundary between the fissure and extrahippocampal cerebrospinal fluid (CSF) (Iglesias et al., 2015) may have also contributed towards its poor reproducibility.

Prior appraisals of the FS5.3 segmentation algorithm noted its inconsistent delineations of the hippocampal head and tail (Yushkevich et al., 2010). This new algorithm – FS6.0 – which relies upon a refined atlas built upon high-resolution *ex vivo* MRI data (Iglesias et al., 2015), appears to reconstruct the hippocampal tail and parts of the hippocampal head (CA1, CA2/3) with a high degree of spatial overlap and test–retest reproducibility. Segmentations of the dentate gyrus have also been criticized in FS5.3, as they appear to mismatch with known anatomical boundaries (Wisse et al., 2014), In FS6.0, the dentate is reconstructed as three individual subregions, namely; the hilar region (CA4), the granule cells (GC-DG) and, partially, the molecular layer. Our study showed stable test–retest reliability in all three subregions.

Prior evaluations of the FS5.3 algorithm also noted that the CA1 is the smallest of the three *cornu ammonis* segmentations (CA1, CA2 & CA3), despite *post-mortem* studies contradictorily indicating that the CA1 is the largest and the CA2&3 are the smallest subfields (Wisse et al., 2014). This neuroanatomical inconsistency may yield misleading clinical interpretations: For example, FreeSurfer-based investigations of the human hippocampal subregions have associated neurological conditions such as MCI or Alzheimer’s disease with atrophy of the CA2&3 (Hanseeuw et al., 2011; Lim et al., 2012), whereas anatomical studies have reported the most profound atrophy in the CA1 (Simic et al., 1997; Rossler et al., 2002). Our findings suggest that this anatomical inconsistency appears to be resolved in FS6.0; the CA1 is now the largest and most reliably reconstructed of the three subfields (see Table 2). Future *in-vivo* investigations of the human hippocampal subregions should therefore prioritize the use of the revised algorithm, FS6.0, as our results show that FS6.0 reliably reproduces eleven major hippocampal subregions across two independent cohorts (QTIM and ADNI-2), despite differences in age, scanning interval and image acquisition method. Clinical findings reported using the algorithm’s predecessor, FS5.3, should be interpreted with caution.

### Between-version agreement and trans-platform reliability: Implications for imaging consortia

International consortia like ENIGMA typically involve large-scale implementation of harmonized segmentation protocols across diverse networks of research laboratories. Many of these laboratories may have already processed their T1-weighted images through older versions (v5.1–5.3) of the FreeSurfer subregion segmentation tool, raising questions about the need to process their data through a new version of the algorithm. Here, we found strong agreement between older (v5.3) and newer (v6.0) versions of the tool for the hippocampal tail, presubiculum and subiculum. However, versions 5.3 and 6.0 produced variable volume estimates for the *cornu ammonis*, fimbria, and hippocampal fissure. These discrepancies were expected, due to the algorithm’s revised definitions of subregional borders (Iglesias et al., 2015). FS6.0 also produced four new subregions with no directly corresponding structures in FS5.3 (the parasubiculum, molecular layer, granule cells of the dentate and HATA). Furthermore, version 6.0 produced slightly more consistent estimates across lower (1.5 T) and higher (3 T) MRI scanner field strengths. Overall, these findings suggest that the latest version of the FreeSurfer subregion segmentation algorithm is a more reliable, versatile and anatomically accurate tool than its predecessors (Iglesias et al., 2015). International consortia such as ENIGMA may benefit by encouraging all participating sites to process their imaging data with the revised segmentation tool (FS6.0). The combination of volume estimates acquired using previous (FS5.3) and revised (FS6.0) algorithms is not recommended.

### Validating the human hippocampal subfields as quantitative phenotypes for genetic mapping

In the second part of this manuscript, we used SOLAR to calculate the heritability of all twelve automatically segmented hippocampal subregions. The greatest genetic effects were observed in larger subregions, particularly within the granule cells of the DG, molecular layer and the hippocampal tail (*h*^2^ = 0.74–0.91). Smaller subregions such as the hippocampal fissure and parasubiculum produced strong but lower heritability estimates (*h*^2^ = 0.56–0.57). This pattern of heritability has previously been reported across the wider collection of subcortical structures, with larger regions (such as the thalamus) showing higher heritability than smaller regions (such as the amygdala) (see Hibar et al., 2015). These heritability fluctuations may be explained by the reduced measurement errors associated with larger segmentations. However, biological factors may also play a role. For example, the *cornu ammonis* is among the earliest brain regions to develop prenatally (Taupin, 2007), whereas the subiculum and CA2 are the first hippocampal subregions to mature postnatally (Jabès et al., 2011). The DG and hippocampal tail show accelerated patterns of neurogenesis after the first postnatal year (Insausti et al., 2010). In adult life, hippocampal neurons continue to proliferate from precursor cells in the DG (Kempermann et al., 2004). Given the early development of the CA subregions (Taupin, 2007) and hippocampal tail (Insausti et al., 2010) and the key memory-processing role of the DG in adulthood (Coras et al., 2014), it is likely that genetic factors significantly influence each region. Total hippocampal volume was also significantly heritable (*h*^2^ = 0.86–0.88) — supporting prior estimates from healthy populations; this further shows the impact of genetic factors on the structure as a whole (den Braber et al., 2013; Swagerman and Brouwer, 2014; van Erp et al., 2004; Wright et al., 2002).

Our main aim here was to identify reliable quantitative phenotypes that can be used in future collaborative genetic mapping efforts. A biomarker must satisfy several explicit criteria before it can be considered an endophenotype (Gottesman and Gould, 2003). First, it should be associated with illness in the population. Structural changes in the hippocampal subregions are implicated in a wide range of brain disorders, from Alzheimer’s disease to epilepsy and schizophrenia (Bartsch, 2012; Sala, 2008). Second, a useful quantitative endophenotype must be heritable. In this study, all major subregions of the hippocampus were highly influenced by additive genetic effects, with heritability estimates ranging from *h*^2^ = 0.56 to *h*^2^ = 0.91. All subregions, with the exception of the hippocampal fissure (which shows inconsistent volume estimates across image acquisition time points and field strengths), could therefore be considered as reliable and robust quantitative phenotypes for future genetic mapping studies.

### Limitations and future directions

In this collaborative investigation, we evaluated a revised version of the FreeSurfer subregion segmentation tool using data collected and analyzed at multiple, independent sites (ADNI-2, QTIM, MPIP and NESDA) at two different field strengths (3 T and 4 T) across large samples of healthy (QTIM, ADNI-2) and affected populations (MPIP, NESDA). We found that the revised algorithm produces heritable and reliable segmentations for eleven human hippocampal subregions, but future users should note some limitations. First, the algorithm has yet to be validated against manual segmentations. A recent quantitative comparison of 21 manual segmentation protocols, including the protocol used to generate manually annotated training data for the revised FreeSurfer algorithm, revealed significant variability among the labels used to define subregions, how boundaries were placed between labels, and the overall extent of the hippocampal formation that is labeled across protocols (Yushkevich et al., 2015). FS6.0 is already a reliable, accessible tool for automated subregion segmentation, but it continues to evolve alongside on-going efforts to harmonize hippocampal subfield protocols (The Hippocampal Subfields Group (HSG), 2014; see hippocampalsubfields.com). As such, it is inevitably subject to revisions as the field develops. Second, although the revised algorithm can segment T1-and T2-weighted images (and their combination; Iglesias et al., 2015), the results presented here are inferred from standard resolution, T1-weighted data only, which is more commonly available across large consortium efforts, such as ENIGMA. Test–retest reliability estimates were extracted using a series of 1.2 mm^3^ and ~0.95 mm^3^ isotropic images, respectively, possibly introducing measurement errors for smaller subregions like the fimbria (mean volume: 98.24 mm^3^), HATA (mean volume: 74.84 mm^3^) and parasubiculum (mean volume: 62.23 mm^3^) (see Table 2). Future versions of the FreeSurfer segmentation algorithm may yield more robust estimates for low resolution data (<1 mm^3^) by combining smaller subfields such as the subiculum and CA2/3. Third, while we observed good reliability between subregion segmentations acquired at 1.5 T and 3 T field strengths, test–retest reproducibility estimates were not established at 1.5 T.

Despite these limitations, the present study supports the utility of eleven automatically segmented hippocampal subregion volumes as quantitative *endophenotypes* for future imaging genetics collaborations. Progressing from macro-level investigations of large brain regions towards more fine-grained maps of specific hippocampal subregions may add more precise localization to GWAS effects. The ENIGMA consortium is now conducting related, finer-grained efforts using diffusion tensor imaging (Jahanshad et al., 2013; Kochunov et al., 2015) and shape analysis (Thompson et al., 2014). Here, we evaluated the automated reconstruction of hippocampal subregion volumes as another useful intermediate biomarker for genome-wide association. As multi-center consortium efforts continue to discover genes associated with brain measures, future quantitative genetic investigations of specific hippocampal subregions may point to a more mechanistic understanding of these genes, and how they affect cognition, behavior and neurological illness.

## Conclusion

The hippocampal formation is one of the most profoundly disrupted brain regions in many neurological and psychiatric illnesses. As the present study illustrates, it is now possible to reconstruct eleven major subregions of the hippocampus using almost fully automated brain imaging methods, to a high degree of accuracy and reliability within and across populations. All eleven subregions are highly influenced by genetic factors. As the field of imaging genetics and large-scale imaging consortia continue to successfully identify genes associated with measures from the living human brain, our results may help these initiatives stratify their traits of interest and better understand the mechanisms of gene action.

## Figures and Tables

**Table 1:** Participant demographics.

Cohort	N	Field strength	Mean age (years + SD)	Age range (years)	Female/male
ADNI-2	163	3T	73.6	56.3–89.1	81/82
QTIM (test–retest)	39	4T	24.03 (3.49)	20.72–27.31	20/19
QTIM (full)^a^	728	4T	22.65 (2.73)	18.1–29.73	465/263
NESDA	221	3T	38.14 (10.33)	18–57	145/76
MPIP	589	1.5 T	48.4 (13.5)	18–87	334/255

‘SD’ = standard deviation, MPIP = Max Planck Institute of Psychiatry, NESDA = Netherlands Study of Depression and Anxiety, ADNI-2 = Alzheimer’s Disease NeuroImaging Initiative, QTIM = Queensland Twins Imaging Study.

a The QTIM cohort included 132 monozygotic twin pairs and 232 dizygotic twin pairs.

**Table 2:** Test–retest intra-class coefficients, dice similarity coefficients and mean volumes for the ADNI-2 and QTIM samples.

Region	Hemi	QTIM 4.0 T Bruker Medscape, N = 39							
		Mean volume (mm^3^)	ICC	CI upper	CI lower	Mean volume (mm^3^)	ICC	CI upper	CI lower
Whole hippocampus	Left	3494.56	.94	.91	.95	3162.66	.88	.79	.94
	Right	3565.37	.97	.95	.98	3250.55	.85	.73	.92
CA1	Left	653.08	.91	.88	.93	574.11	.86	.75	.92
	Right	676.32	.94	.91	.95	602.92	.89	.80	.94
Molecular layer	Left	572.70	.93	.90	.95	515.64	.86	.75	.92
	Right	593.24	.96	.94	.97	528.06	.88	.78	.93
Hippocampal tail	Left	510.04	.93	.91	.95	496.55	.83	.69	.90
	Right	511.88	.93	.91	.95	523.04	.72	.53	.84
Subiculum	Left	407.45	.91	.88	.93	388.12	.80	.65	.89
	Right	411.34	.94	.92	.96	388.72	.86	.74	.91
Granule cells in the molecular layer of the DG (GC-ML-DG)	Left	315.69	.91	.88	.93	277.84	.78	.62	.88
	Right	326.69	.94	.92	.96	288.63	.78	.62	.88
Presubiculum	Left	297.3	.9	.86	.92	291.6	.89	.80	.94
	Right	291.18	.92	.89	.94	276.73	.65	.42	.80
CA4	Left	271.82	.9	.87	.93	241.75	.75	.58	.86
	Right	283.69	.92	.89	.94	251.44	.77	.60	.87
CA3	Left	227.13	.88	.85	.91	203.38	.78	.62	.88
	Right	239.38	.93	.91	.95	220.03	.82	.68	.90
Hippocampal fissure	Left	159.59	.88	.84	.91	157.05	.80	.64	.88
	Right	162.1	.9	.86	.92	164.92	.70	.51	.84
Fimbria	Left	99.83	.9	.88	.93	56.77	.80	.65	.89
	Right	96.64	.89	.86	.92	52.67	.86	.75	.93
Hippocampal-amygdaloid transition area (HATA)	Left	77.19	.79	.73	.85	56.16	.50	.23	.70
	Right	72.48	.78	.71	.83	56.69	.64	.41	.79
Parasubiculum	Left	62.23	.81	.75	.86	60.69	.74	.55	.85
	Right	62.24	.75	.67	.80	61.06	.68	.46	.81

**Table 3:** Intra-class correlation coefficients for between-version agreement (MPIP cohort, N = 589, 3 T).

Region (bilateral)	Version 5.3 →							
Version 6.0 ↓	Tail	CA1	CA2_3	CA4_DG	Fimbria	Fissure	Presubiculum	Subiculum
Tail	0.778	–	–	–	–	–	–	–
CA1	–	0.645	0.817	0.872	–	–	–	–
CA3	–	0.607	0.383	0.594	–	–	–	–
CA4	–	0.673	0.405	0.661	–	–	–	
Fimbria	–	–	–	–	0.780	–	–	–
Fissure	–	–	–	–	–	0.716	–	
Presubiculum	–	–	–	–	–	–	0.797	–
Subiculum	–	–	–	–	–	–	–	0.857

**Table 4:** Intra-class correlation coefficients for between-version agreement (ADNI-2 cohort, N = 163, 3 T).

Region (bilateral)	Version 5.3 →							
Version 6.0 ↓	Tail	CA1	CA2_3	CA4_DG	Fimbria	Fissure	Presubiculum	Subiculum
Tail	0.839	–	–	–	–	–	–	–
CA1	–	0.661	0.774	0.901	–	–	–	–
CA3	–	0.523	0.344	0.567	–	–	–	–
CA4	–	0.598	0.372	0.633	–	–	–	–
Fimbria	–	–	–	–	0.805	–	–	–
Fissure	–	–	–	–	–	0.628	–	–
Presubiculum	–	–	–	–	–	–	0.825	–
Subiculum	–	–	–	–	–	–	–	0.833

**Table 5:** Intra-class correlation coefficients for between-version agreement (NESDA cohort, N = 221, 3 T).

Region (bilateral)	Version 5.3 →							
Version 6.0 ↓	Tail	CA1	CA2_3	CA4_DG	Fimbria	Fissure	Presubiculum	Subiculum
Tail	0.778	–	–	–	–	–	–	–
CA1	–	0.698	0.694	0.856	–	–	–	–
CA3	–	0.679	0.334	0.545	–	–	–	–
CA4	–	0.729	0.343	0.592	–	–	–	–
Fimbria	–	–	–	–	0.758	–	–	–
Fissure	–	–	–	–	–	0.321	–	–
Presubiculum	–	–	–	–	–	–	0.783	–
Subiculum	–	–	–	–	–	–	–	0.815

**Table 6:** DICE coefficients for between-version spatial overlap in the ADNI-2, NESDA and MPIP cohorts.

	Tail	CA1	CA2_3	CA4_DG	Fimbria	Fissure	Presubiculum	Subiculum	Whole
ADNI-2	0.68	0.40	0.30	0.50	0.45	0.30	0.60	0.56	0.85
NESDA	0.67	0.39	0.28	0.51	0.53	0.33	0.57	0.55	0.82
MPIP	0.70	0.40	0.30	0.49	0.51	0.32	0.62	0.58	0.83

**Table 7:** Trans-platform reliability across 1.5 T and 3 T field strengths, using estimates extracted from using FreeSurfer v5.3 and v6.0 (MPIP cohort, N = 10, 3 T).

Region (bilateral)	ICC (FS 5.3)	ICC (FS 6.0)
Whole hippocampus	0.855	0.960
CA1	0.725	0.915
CA2 3	0.856	0.871
CA4_DG	0.892	0.792
Fimbria	0.720	0.721
Fissure	0.465	0.575
Presubiculum	0.818	0.853
Subiculum	0.866	0.858
Tail	0.875	0.863
Parasubiculum	–	0.659
GC-ML-DG	–	0.828
Molecular_layer_HP	–	0.932
HATA	–	0.801

Median cross-platform reliability ICC across values = 0.855 (FreeSurfer 5.3), 0.853 (FreeSurfer 6.0).

**Table 8:** Heritability estimates for hippocampal subfield volumes, calculated using FreeSurfer v6.0 (QTIM cohort, N = 728, 4 T).

Region	QTIM		
	*h*^2^	Std. error	*p*-Value
Hippocampal fissure	0.56	0.06	1.90 × 10^−14^
Parasubiculum	0.57	0.05	6.16 × 10^−17^
Fimbria	0.64	0.05	3.06 × 10^−19^
HATA	0.67	0.04	2.76 × 10^−24^
CA3	0.75	0.03	4.23 × 10^−33^
Subiculum	0.76	0.03	5.02 × 10^−32^
CA4	0.79	0.03	1.27 × 10^−38^
Presubiculum	0.72	0.04	6.80 × 10^−30^
CA1	0.84	0.02	2.54 × 10^−47^
Granule cells of DG	0.82	0.03	5.66 × 10^−41^
Molecular layer of DG	0.85	0.02	2.56 × 10^−49^
Whole hippocampus	0.88	0.01	1.19 × 10^−54^
Hippocampal tail	0.84	0.02	3.28 × 10^−44^
